# Establishing Reliable miRNA-Cancer Association Network Based on Text-Mining Method

**DOI:** 10.1155/2014/746979

**Published:** 2014-04-10

**Authors:** Lun Li, Xingchi Hu, Zhaowan Yang, Zhenyu Jia, Ming Fang, Libin Zhang, Yanhong Zhou

**Affiliations:** ^1^Hubei Bioinformatics and Molecular Imaging Key Laboratory, Huazhong University of Science and Technology, Wuhan 430074, China; ^2^Biomedical Engineering Department, College of Life Science and Technology, Huazhong University of Science and Technology, Wuhan, Hubei 430074, China; ^3^Department of Statistics, The University of Akron, Akron, OH 44325, USA; ^4^Department of Family & Community Medicine, Northeast Ohio Medical University, Rootstown, OH 44272, USA; ^5^Guizhou Provincial Key Laboratory of Computational Nano-Material Science, Guizhou Normal College, Guiyang 550018, China

## Abstract

Associating microRNAs (miRNAs) with cancers is an important step of understanding the mechanisms of cancer pathogenesis and finding novel biomarkers for cancer therapies. In this study, we constructed a miRNA-cancer association network (miCancerna) based on more than 1,000 miRNA-cancer associations detected from millions of abstracts with the text-mining method, including 226 miRNA families and 20 common cancers. We further prioritized cancer-related miRNAs at the network level with the random-walk algorithm, achieving a relatively higher performance than previous miRNA disease networks. Finally, we examined the top 5 candidate miRNAs for each kind of cancer and found that 71% of them are confirmed experimentally. miCancerna would be an alternative resource for the cancer-related miRNA identification.

## 1. Introduction

MicroRNAs (miRNAs) are a large class of small noncoding RNAs [1] known to be functionally involved in a wide range of biological processes including embryo development, cell growth, differentiation, apoptosis, and proliferation [2–5]. Recently, it has been found that miRNAs play important roles in human tumor genesis and many of them have also been applied as novel biomarkers for cancer therapies [6–11], which attracts more and more efforts in revealing the complex associations between miRNAs and cancers. However, the existing literature usually focused on the relationship between several miRNAs and a specific cancer, leaving the comprehensive miRNA-cancer network unrevealed. Therefore, fully uncovering the associations between miRNAs and cancers would be extremely interesting and valuable for identifying cancer-related miRNA and understanding the mechanisms behind.

To this aim, the manually collected miRNA-disease association databases HMDD [12] and miR2Disease [13] have been established. At present, these manually created miRNA-disease networks have been used to predict disease-related miRNAs [14–16] and  achieved relatively high accuracies, opening opportunity of prioritizing miRNAs  with bioinformatics methods.

However, thousands of papers on miRNA and cancer researches are published each year, making it difficult to manually check papers. On the other hand, automatic text-mining methods are needed to extract reliable miRNA-disease associations [17] from the increasing database.

In this paper, we collected 1,018 associations between 226 miRNA families and 20 common cancers by extracting from more than 7.1 million publications with an automatic text-mining method. All these relationships have been recorded in a database named miCancerna, which can be freely assessed at http://micancerna.appspot.com/. We further constructed a miRNA-cancer general view on top 5% significant associations for visualizing the roles of miRNAs in different cancers and prioritized the cancer-related miRNAs using the random walk with restart algorithm (RWRA) [14] on miRNA-cancer network built on the data in miCancerna. By analyzing the top 5 associated miRNAs of 20 cancers according to Fisher's exact tests, we found experimental evidence for 71% of these miRNA-cancer relationships, and the rest might be candidate cancer-related miRNAs for further experimental validation. The constructed miRNA-cancer network would be extremely valuable for comprehensively understanding the mechanisms of cancers and identifying cancer-related miRNA genes.

## 2. Materials and Methods

### 2.1. Collecting Resource Literature

We collected the abstracts from NCBI's MEDLINE database as our target literature resource. MEDLINE is a comprehensive database containing the abstracts of millions of articles in biomedical area. Since a large number of papers are not fully accessible in the PubMed database, we only consider the abstracts for the papers, which are always available.

In 2000, Reinhart et al. [18] identified the second miRNA, and thereafter researchers began to pay attention to the importance of miRNAs. Therefore, we mainly focus on the papers that have been published in 2000 and after. In total, 7,207,066 abstracts were retrieved and then screened using keywords, such as “Humans” or “Animals,” within the PubMed search for eliminating plant and virus miRNAs in the following text-mining analysis. This filtration yielded 5,606,308 paper abstracts.

Currently, the 20 most common cancers reported by National Cancer Institute (http://www.cancer.gov/) are considered in our study, including leukemia, lung cancer bladder cancer, brain cancer, breast cancer, cervix cancer, colorectal cancer, esophageal cancer, kidney cancer, liver cancer, melanoma, myeloma, non-Hodgkin lymphoma, oral cancer, ovarian cancer, pancreatic cancer, prostate cancer, stomach cancer, thyroid cancer, and uterine cancer. The abstracts are individually marked with cancer types by the following steps: first, we mapped each cancer type to its corresponding MeSH (medical subject headings) term(s), the U.S. National Library of Medicine's controlled vocabulary that are manually assigned for articles archived in MEDLINE describing their subject matters, and then compiled a list of standard names of each type of cancer. Subsequently, we searched each article abstract for the MeSH annotations. The abstracts with MeSH terms in our cancers name list are marked with the corresponding cancer and selected for the following text-mining processing.

### 2.2. Establishing miRNA-Cancer Networks by Text-Mining Method

With the selected abstracts, we firstly established relationships between miRNAs and cancers by a text-mining method. The associations between miRNAs and cancers were estimated based on the cooccurrence assumption, which is the fundamental assumption in the field of text-mining and can be used to infer whether two terms are associated or not. In our case, if a particular miRNA appears in the abstracts marked by a specific cancer frequently, we can reasonably assume that they cooccurred and tend to be related. To establish the associations between miRNAs and cancers, we detect the appearance of miRNAs in the abstracts marked by cancer types. In this study, the regular expression was applied to match miRNA names against the texts with the following steps. (1) miRNAs (such as “miR-1” and “miR-2”) were firstly extracted from the abstracts with the nomenclature of a “miR” prefix accompanied by a unique identifying number [19]. (2) Following the conventions, a prefixed species/state identifier can be added (e.g., “hsa-miR-1” in* Homo sapiens* and “pre-miR-1” for a precursor) and additional suffixes can be given to indicate loci or variant (e.g., “miR-1a-1”) [20]. (3) The regular expression was also designed for the variants of some miRNAs, such as “lin-4” and “let-7.” (4) Abbreviations for more than one miRNA are also recognized by the regular expression, for example, “miR-221/222” and “miR-15 & -16.”

The significance levels of the associations of the miRNAs and the cancers extracted from the marked abstracts were estimated by one-sided Fisher's exact tests [21]. For a pair of the miRNA *M* and the cancer *C*, the *P* value of Fisher's exact test is calculated based on hypergeometric distribution, as follows: $P=\left(\begin{array}{c} a+b\\ a \end{array}\right)\left(\begin{array}{c} c+d\\ c \end{array}\right)/\left(\begin{array}{c} n\\ a+c \end{array}\right)=\left(a+b\right)!\left(c+d\right)!\left(a+c\right)!\left(c+d\right)!/\left(a!b!c!d!n!\right)$, where *n* is denoted as the total number of papers included in the text-mining analysis, *a* stands for the number of papers with both the miRNA *M* and the cancer *C* in the abstracts, *b* and *c* represent, respectively, the number of abstracts containing one termand excluding the other, and *d* is the number of papers with neither of the terms. The top 5% miRNA-cancer associations with the minimum *P* value are considered as significant and were used to generate the general view for miRNA-cancer network. The miRNA-cancer network is a bipartite network composed by miRNA nodes and cancer nodes. Each edge in miCancerna connects a miRNA and one of its corresponding cancers.

### 2.3. Text-Mining Quality Check

We first queried PubMed with “MIR or MIRN or MIRNA or MICRORNA” and randomly picked up 100 MEDLINE abstracts with at least one miRNA identifier from the querying result as our evaluating data. We then investigated the reliability of detecting miRNAs in texts using the *F*-measure, which is the harmonic mean of two other measures, recall and precision, as follows:

(1) $$\begin{array}{c} \text{Recall}=\frac{\text{TP}}{\text{TP}+\text{FN}},\\ \text{Precision}=\frac{\text{TP}}{\text{TP}+\text{FP}},\\ F\text{-measure}=\frac{2\times \text{Recall}\times \text{Precision}}{\text{Recall}+\text{Precision}}, \end{array}$$

where TP, FP, and FN are the number of true positives, false positives, and false negatives, respectively.

### 2.4. Random Walk with Restart Method

Based on the network constructed by the data from miCancerna, a random walk with restart (RWRA) method is applied to prioritize cancer-related miRNAs.

RWRA is one of the random walk models widely used in disease gene discovery [22]. It simulates a random walker's moves in a given network and the walker moves from a current node to a direct neighboring node or restart with a training node with the probability (*α*). The movement given out by RWRA is defined as follows:

(2) $$\begin{array}{c} P_{t+1}=\left(1-\alpha \right)MP_{t}+\alpha P_{0}, \end{array}$$

where *M* is a column-normalized adjacency matrix representing the given network. In this case, each nonzero node in *M* stands for a certain association between a miRNA and a cancer, and these nodes are taken as seeds. *P*_*t*_ is a vector representing the probabilities of the walker at each node at time *t*, and *P*_0_ is the initial probability vector in which training nodes are equally assigned 1/*N* (*N* is the number of seeds) while others are 0. The process is iterated until *P* reaches a stable status when the difference between *P*_*t*+1_ and *P*_*t*_ (measured by *L*1 norm) is less than a threshold value (10^−6^ in this study). The stable probability is defined as *P*_*∞*_. The candidate nodes are then ranked in descending order according to *P*_*∞*_.

### 2.5. Leave-One-Out Cross-Validation

The performance of cancer-related miRNA prioritization by random walk with restart algorithm through miCancerna could be evaluated by calculating the area under the ROC through the leave-one-out cross-validation. For each training node, we took it as a candidate node and randomly picked 20 miRNAs not belonging to the same cancer as testing nodes and then prioritized them as above. For each threshold, the sensitivity (SN) and specificity (SP) are defined as follows:

(3) $$\begin{array}{c} \text{SN}=\frac{\text{TP}}{\text{TP}+\text{FN}},\\ \text{SP}=\frac{\text{TN}}{\text{FP}+\text{TN}}, \end{array}$$

where TP (true positive) is the number of training nodes with rank above the threshold, FN (false negative) is the number of training nodes with rank under the threshold, TN (true negative) is the number of testing nodes with rank under the threshold, and FP (false positive) is the number of test nodes with rank above the threshold. The ROC curve shows the relationship between SN and 1-SP, and the AUC means the area under the ROC curve.

## 3. Result and Discussion

### 3.1. Online Resource for miRNA-Cancer Network

In the first release, miCancerna records 1,018 associations between 226 miRNA families and 20 common cancers extracted from 7.2 million papers. Now all the data that miCancerna refers to can be freely assessed at http://micancerna.appspot.com/, including the associations, the supporting papers, and significant levels for each association. miCancerna will be updated periodically.

To check the text-mining quality, we randomly picked up 100 MEDLINE abstracts that contained at least one miRNA identifier from the search results by querying MEDLINE with “MIR or MIRN or MIRNA or MICRORNA.” A total of 739 miRNA identifiers were manually recognized in the texts of evaluating data, while our regular expression correctly matched 735 of them (true positive, TP), miscalled 2 (false positive, FP), and missed 4 (false negative, FN). So the miRNA annotation gained recall of 0.9946, precision of 0.9973, and *F*-measure of 0.9959, which demonstrated a fairly high reliability of our regular expression.

According to these comparison results, we concluded that miCancerna is a high-quality resource of miRNA-cancer associations.

### 3.2. miRNA-Cancer Network Visualization

**Figure 1:**
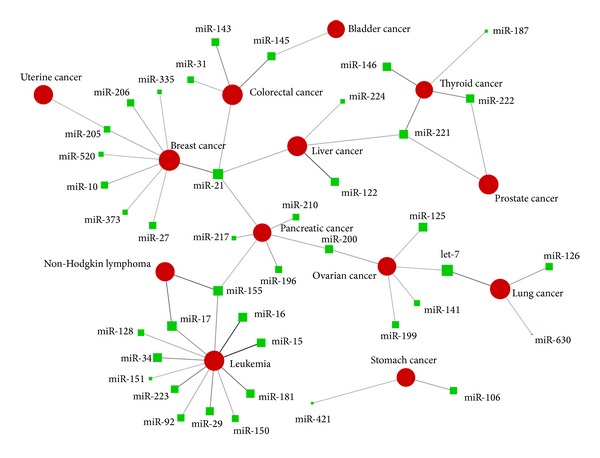
Network illustrated significant associations of miRNAs and cancers. Red circles and green squares represent cancers and miRNAs, respectively, with different sizes according to the number of corresponding annotated papers (logarithmic). Each link represents a miRNA-cancer association with colour and width according to the strength of relationship.

To reveal the roles of miRNA in different cancers, we constructed a bipartite network with the top 5% associations based on Fisher's exact test *P* values in miCancerna, consisting of 40 miRNA families and 13 types of cancers (Figure 1). In this bipartite network, miRNAs are only connected to cancers and cancers are only connected to miRNAs. The miRNA-cancer network was visualized with Pajek (http://vlado.fmf.uni-lj.si/pub/networks/pajek/). It is interesting to find that almost all these cancers (except the stomach cancer) can be connected via miRNAs, which indicated that different cancers might share common pathogenic components regulated by  these interconnected miRNAs, while stomach cancer may be different with others.

As shown in Figure 1, miRNAs may have different involvements in cancers. Some miRNAs are specifically associated with a specific cancer. For example, miR-15 and miR-16 are tendentiously related to leukemia, and miR-122 is almost exclusively associated with liver cancer. These miRNAs may be used as biomarker candidates for diagnosis and efficacy of therapies for corresponding cancers. By contrast, some miRNAs tend to be associated with various cancers. One example is miR-21, which is shown to significantly associate with breast cancer, colorectal cancer, liver cancer, and pancreatic cancer, indicating that target genes of miR-21 might play critical roles in tumor formation.

It is interesting that four miRNA-cancer associations in top 10 (Table 1) are miRNA-leukemia associations, and 28.6% (12) of significant associations were related to leukemia, which makes leukemia the most miRNA-related cancer. Similarly, 8 (19.0%) miRNA families were related to breast cancer in significant miRNA-cancer associations. Furthermore, we found that miR-21 is the most cancer-related miRNA, which is associated with 4 (30.77%) different cancers in significant associations (breast cancer, pancreatic cancer, liver cancer, and colorectal cancers), indicating that miR-21 may be involved in an important pathway in cancer formation.

### 3.3. Prioritization of Cancer-Related miRNAs

**Figure 2:**
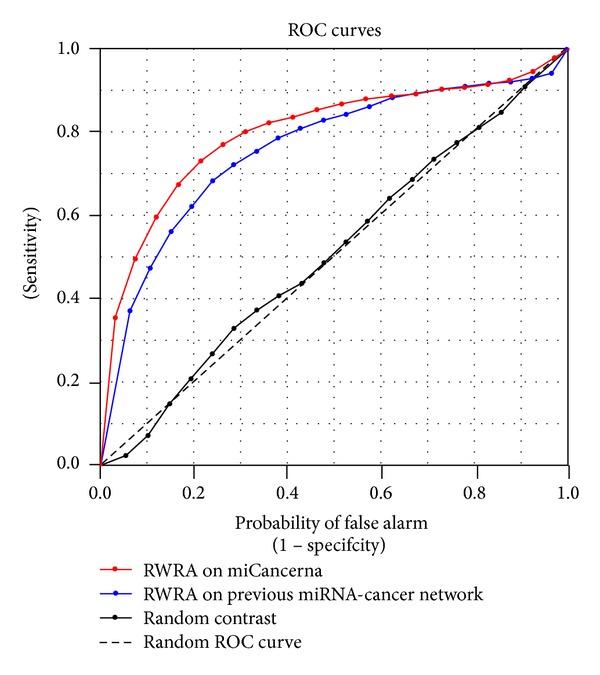
ROC curves for RWRA on miCancerna and previous miRNA-cancer network.

**Figure 3:**
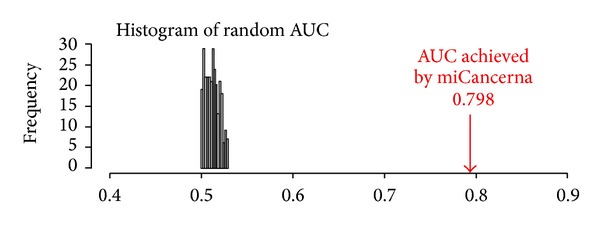
Distribution of random AUC for miCancerna.

We applied RWRA on the network established by miCancerna to prioritize candidate cancer-related miRNAs, and the performance is evaluated by leave-one-out cross-validation. With a restart probability alpha of 0.9, the AUC of ROC curve can reach 0.798 (Figure 2), while the AUC of 1 stands for the perfect performance and AUC of 0.5 indicates the random performance. The performances with different restart probabilities are showed in Table 2. The AUC improves as alpha increases, but the variation is small. To rule out the possibility that the performance of miCancerna is achieved by chance, a permutation test with 300 runs was performed. For each run, the seeds are randomly selected from the candidate nodes. The average AUC of random permutations obtained by leave-one-out cross validation is 0.513, and the distribution of the random permutation AUCs is shown in Figure 3. It is obvious that there is significant difference between the AUC achieved by miCancerna and the random permutations, which supports that the miCancerna reveals the real involvement of miRNAs in cancer biology.

The top 5 potential miRNAs of each cancer are presented in Table 3, among which 71% have been evaluated by experimental evidence in dbDEMC [23] or literatures published after miCancerna. The performance of cancer-related miRNA prioritization demonstrates the reliability of miCancerna. Moreover, the top predicted miRNAs may be the potential cancer-related miRNAs for further study.

### 3.4. Comparison with Similar Databases

We made comparisons with similar database or networks. First we compared the data involved in miCancerna and the manual checking database miR2Disease on the number of evidence papers. For most cancers, miCancerna provides much more evidence papers than miR2Disease (Table 4). Second, we compared the prediction performance of RWRA on miCancerna with the miRNA-cancer network used in RWRMDA [14], which was built based on HMDD, a manual database. The ROC curves for both networks are showed in Figure 2. According to the result of leave-one-out cross-validation, the network used in RWRMDA achieved AUC of 0.763, which is lower than 0.797 achieved by miCancerna.

These results indicate that miCancerna provides an alternative resource of miRNA-cancer associations.

## 4. Conclusion

In this study, we constructed a reliable miRNA-cancer network based on text-mining method, which is stored in the database miCancerna. In current release, there are 1,018 associations between 226 miRNA families and 20 common cancers. According to our test result, the miCancerna provides a reliable and comprehensive resource of miRNA-cancer associations, which can be further used in the identification of cancer-related miRNAs.

For future development, we plan to consider more types of cancers, add regulation information to the miRNA-cancer associations, and integrate miCancerna into other related databases, such as MISIM [24], the human miRNA functional similarity and functional network.

## Figures and Tables

**Table 1:** Top 10 associates between miRNAs and cancers.

miRNA	Cancer	Papers	*P* value
miR-15	Leukaemia	35	6.804 × 10^−43^
miR-16	Leukaemia	33	5.028 × 10^−36^
miR-122	Liver cancer	22	9.742 × 10^−26^
miR-181	Leukaemia	23	3.142 × 10^−25^
miR-155	Non-Hodgkin lymphoma	22	7.393 × 10^−22^
Let-7	Lung cancer	34	1.110 × 10^−19^
miR-223	Leukaemia	16	1.987 × 10^−18^
miR-17	Non-Hodgkin lymphoma	19	3.772 × 10^−18^
miR-21	Breast cancer	31	1.659 × 10^−16^
miR-221	Thyroid cancer	11	1.607 × 10^−14^

**Table 2:** AUC value under different alpha.

Alpha	0.1	0.2	0.3	0.4	0.5	0.6	0.7	0.8	0.9
AUC	0.7952	0.7973	0.7974	0.7978	0.7981	0.7981	0.7983	0.7983	0.7984

**Table 3:** Top 5 potential miRNAs of 20 cancers.

Bladder cancer		Brain cancer		Breast cancer		Cervix cancer	
miRNAs	Confirm	miRNAs	Confirm	miRNAs	Confirm	miRNAs	Confirm
miR-15	Null	let-7	Ref. [25]	miR-143	dbDEMC	let-7	Null
miR-34	Ref. [26]	miR-145	Ref. [27]	miR-223	dbDEMC	miR-221	Null
miR-16	Ref. [26]	miR-16	Ref. [28]	miR-203	dbDEMC	miR-17	Ref. [29]
miR-146	Ref. [30]	miR-155	Ref. [31]	miR-194	dbDEMC	miR-125	Null
miR-155	Ref. [30]	miR-143	Ref. [28]	miR-100	dbDEMC	miR-222	Null

Colorectal cancer		Esophageal cancer		Kidney cancer		Leukemia	
miRNAs	Confirm	miRNAs	Confirm	miRNAs	Confirm	miRNAs	Confirm

miR-221	dbDEMC	miR-17	dbDEMC	miR-125	dbDEMC	miR-200	Ref. [32]
miR-146	dbDEMC	miR-222	dbDEMC	miR-222	dbDEMC	miR-205	Null
miR-29	dbDEMC	miR-15	dbDEMC	miR-146	dbDEMC	miR-193	Null
miR-199	dbDEMC	miR-125	dbDEMC	miR-16	dbDEMC	miR-9	Ref. [33]
miR-193	Null	miR-200	dbDEMC	miR-143	dbDEMC	miR-31	Ref. [34]

Liver cancer		Lung cancer		Melanoma		Myeloma	
miRNAs	Confirm	miRNAs	Confirm	miRNAs	Confirm	miRNAs	Confirm

miR-205	Null	miR-23	dbDEMC	miR-21	Ref. [35]	miR-145	Null
miR-27	dbDEMC	miR-148	dbDEMC	miR-145	Ref. [36]	miR-200	Null
miR-124	Ref. [37]	miR-27	dbDEMC	miR-26	Null	miR-221	Ref. [38]
miR-520	dbDEMC	miR-203	dbDEMC	miR-143	Ref. [36]	miR-34	Null
miR-203	Ref. [39]	miR-520	dbDEMC	miR-126	Ref. [35]	miR-205	Null

Non-Hodgkin lymphoma		Oral cancer		Ovarian cancer		Pancreatic cancer	
miRNAs	Confirm	miRNAs	Confirm	miRNAs	Confirm	miRNAs	Confirm

miR-200	dbDEMC	miR-15	Null	miR-26	Null	miR-16	Ref. [40]
miR-205	dbDEMC	miR-205	Ref. [41]	miR-181	Null	miR-125	Ref. [42]
miR-126	dbDEMC	miR-10	Ref. [43]	miR-143	Ref. [44]	miR-26	Null
miR-224	dbDEMC	miR-182	Null	miR-10	Null	miR-126	Ref. [45]
miR-23	dbDEMC	miR-20	Null	miR-23	Null	miR-181	Ref. [40]

Prostate cancer		Stomach cancer		Thyroid cancer		Uterine cancer	
miRNAs	Confirm	miRNAs	Confirm	miRNAs	Confirm	miRNAs	Confirm

miR-155	dbDEMC	miR-155	Ref. [46]	miR-15	Null	miR-17	dbDEMC
miR-29	Null	miR-29	Null	miR-34	Null	miR-222	dbDEMC
miR-30	dbDEMC	miR-30	Null	miR-145	Ref. [47]	miR-224	dbDEMC
miR-10	dbDEMC	miR-10	Ref. [48]	miR-16	Null	miR-30	dbDEMC
miR-199	dbDEMC	miR-199	Null	miR-205	Ref. [49]	miR-106	dbDEMC

“Null” means we did not find experimental evidence.

**Table 4:** Number of evidence papers comparing with miR2Diease.

Cancer types	miCancerna	miR2Disease	Increase
Bladder cancer	14	11	27.27%
Brain cancer	35	3	1067%
Breast cancer	137	58	136.2%
Cervix cancer	11	4	175%
Colorectal cancer	81	39	107.7%
Esophageal cancer	16	7	128.6%
Kidney cancer	14	4	250.0%
Leukemia	146	45	224.4%
Liver cancer	99	39	153.8%
Lung cancer	112	37	202.7%
Melanoma	21	9	133.3%
Myeloma	9	3	200.0%
Non-Hodgkin lymphoma	62	13	376.9%
Oral cancer	19	0	—
Ovarian cancer	47	18	161.1%
Pancreatic cancer	47	16	193.8%
Prostate cancer	61	19	221.1%
Stomach cancer	48	16	200.0%
Thyroid cancer	21	9	133.3%
Uterine cancer	28	5	460.0%
